# 17-β-Estradiol and Progesterone as Efficient Predictors of Survival in Older Women Undergoing Hip Fracture Surgery

**DOI:** 10.3389/fmed.2020.00345

**Published:** 2020-08-07

**Authors:** Yinwang Zhang, Zhen Xu, Jingyuan Zhang, Jie Tang, Fuhai Liu, Yunxiao Song, Jie Chen

**Affiliations:** ^1^Department of Orthopedics, Shanghai Xuhui Central Hospital, Shanghai, China; ^2^Department of Respiratory Medicine, Shanghai Jiao Tong University Affiliated Sixth People's Hospital, Shanghai, China; ^3^Department of Clinical Laboratory, Shanghai Xuhui Central Hospital, Shanghai, China

**Keywords:** 17-β-estradiol, hip fracture, mortality, older women, progesterone

## Abstract

**Objective:** Sex hormones have been linked to fractures in older women. The purpose of this present study was to investigate the prognostic impact of preoperative sex hormone levels on 30-day mortality in older women undergoing hip fracture surgery.

**Patients and Methods:** A total of 157 female subjects with hip fractures were eligible for the study conducted from January 2010 to December 2019. The serum levels of sex hormones [follicle-stimulating hormone, prolactin, progesterone, testosterone, luteinizing hormone, and 17-β-estradiol (E2)] were measured at admission. To evaluate the prognostic significance of sex hormone levels, Cox proportional hazard models and Kaplan–Meier analyses were applied.

**Results:** Of the 157 subjects, 13 (8.28%) deceased within 30 days. The deceased subjects had lower progesterone (*P* = 0.021) and E2 (*P* < 0.001) levels than the surviving group. Higher progesterone (HR = 0.168, 95% CI = 0.037–0.673) and E2 (HR = 0.857, 95% CI = 0.690–0.968) levels were the key protective factors for 30-day mortality in older women undergoing hip fracture surgery. Survival analysis showed that subjects with lower E2 or/and progesterone levels had a significantly higher percentage of 30-day mortality (log-rank test, *P* < 0.05).

**Conclusion:** E2 and progesterone might be effective predictors of 30-day mortality in older women undergoing hip fracture surgery.

## Introduction

Hip fracture is one of the most common causes of injuries in the elderly, and the rapid global increase in the number of hip fracture patients has placed a huge burden on their caregivers and communities (1). Approximately 6.3 million people worldwide will suffer from hip fractures by 2050 (2). Furthermore, ~33% of women who survive to the age of 90 years will experience a hip fracture by that age (3). Even with treatment, the 30-day mortality of hip fracture patients is up to 11% (4). Various factors (5–8), including advanced age, potentially modifiable conditions such as anemia, electrolyte derangements, and inflammation, are associated with the increased risk of mortality after a hip fracture.

Numerous studies support the association between higher sex hormone levels and lower risk of hip fractures (9–12). For example, Cummings et al. found that women with both undetectable serum estradiol concentrations and serum sex hormone-binding globulin concentrations of 1 μg/dL or more had a relative risk of 6.9 for hip fracture (95% CI = 1.5–32.0) (9). Cauley et al. reported that lower serum 17-β-estradiol (E2) levels might be associated with higher risks of fractures during menopausal transition (12). Evidence from cross-sectional studies shows that estrogen plays a major—and likely dominant—role in bone metabolism (13, 14). Furthermore, estrogen is associated with decreased inflammatory response because it reduces cytokine levels in the spinal cord (15). Bone metabolism and inflammatory response are both associated with the clinical outcome after a hip fracture surgery. However, whether sex hormone concentration is associated with 30-day mortality in patients undergoing hip fracture surgery is still unknown.

We hypothesized that decreased sex hormone concentrations led to a hyperinflammatory state and block osteogenesis, leading to poor clinical outcomes in postmenopausal women after hip fracture surgery. To test this hypothesis, we performed a cohort study to assess whether the levels of baseline sex hormones could be used to predict 30-day mortality in older women undergoing hip fracture surgery.

## Patients and Methods

### Study Population

The study was approved by the Ethics Committee of the Shanghai Xuhui Central Hospital, Shanghai, China, and was conducted according to the Declaration of Helsinki. Subjects were recruited from the Department of Orthopedics, Shanghai Xuhui Central Hospital, from January 2010 to December 2019 according to the inclusion criteria listed in the section that follows. Informed consent was obtained from all subjects.

### Inclusion Criteria

**Figure 1:**
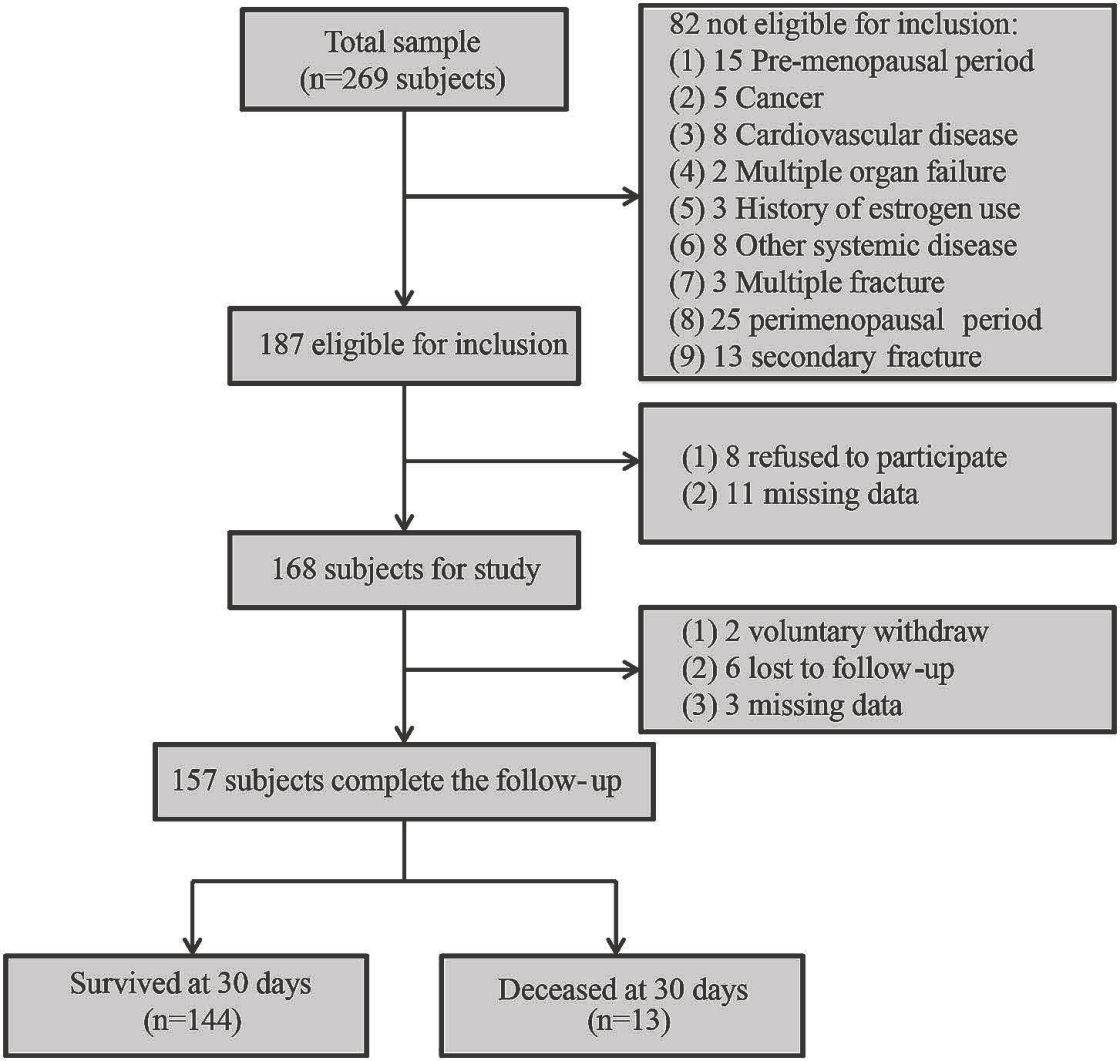
The study cohort flow diagram.

To recruit hip fracture patients, 269 older women with hip fracture who visited the Department of Orthopedics, Shanghai Xuhui Central Hospital, from January 2010 to December 2019 were enrolled. Of these, 112 patients were excluded, resulting in a final sample of 157 patients. Figure 1 presents the study cohort flow diagram.

The following were the inclusion criteria: (1) the subjects had hip fracture proven via imagological examination; (2) the minimum age of the subjects was 55 years; (3) blood samples were preoperatively collected; (4) complete clinical, laboratory, imaging, and follow-up data were collected; (5) the subjects were selected from in-patients; (6) the subjects were postmenopausal women; (7) the subjects were free from systemic diseases; and (8) the subjects were undergoing hip fracture surgery (internal fixation or joint replacement).

### Data Collection

Medical examinations were performed for all subjects at the Shanghai Xuhui Central Hospital. Clinical and demographic information was obtained from the medical data platform of the hospital. These data included information on the following: age, sex, height, weight, diabetes, hypertension, blood pressure, medical history, date of diagnosis, and date of death. As part of the standard care at the Shanghai Xuhui Central Hospital, peripheral blood samples were routinely collected at admission.

During the follow-up period, the subjects were interviewed by a trained assistant until death events occurred. Survival status, disease progression, and date of death were recorded. The last follow-up was conducted on January 30, 2020.

### Laboratory Analysis

Blood samples (4 mL) for routine blood examination were collected in a tube via standard venipuncture in the antecubital fossae (anterior elbow veins). The samples were centrifuged for 10 min at 1,509 g. The blood samples were processed within 1 h of collection. Laboratory testing was performed at the Department of Clinical Laboratory, Shanghai Xuhui Central Hospital. Serum levels of sex hormones (follicle-stimulating hormone, prolactin, progesterone, testosterone, luteinizing hormone, and E2) were measured via sensitive electrochemiluminescence detection using the commercially available Mindray CL-6000M kit (Shenzhen, China). The inter- and intra-assay coefficients of variation, respectively, were 8.6 and 5.7% for E2, 4.6 and 2.8% for prolactin, 8.2 and 3.3% for progesterone, 10.8 and 5.2% for follicle-stimulating hormone, 2.0 and 1.1% for luteinizing hormone, and 4.1 and 2.5% for testosterone. The mean measurement bias for these hormones averaged 2.4% (E2), 1.8% (prolactin), 2.4% (progesterone), 2.8% (follicle-stimulating hormone), 1.6% (luteinizing hormone), and 2.5% (testosterone). Further, their measurement range was 5–3,000 pg/mL (E2), 0.047–470 ng/mL (prolactin), 0.05–60 ng/mL (progesterone), 0.2–200 mIU/mL (follicle-stimulating hormone), 0.1–200 mIU/mL (luteinizing hormone), and 0.025–15 ng/mL (testosterone). Their lower limit of detection averaged to 3 pg/mL (E2), 0.040 ng/mL (prolactin), 0.03 ng/mL (progesterone), 0.1 mIU/mL (follicle-stimulating hormone), 0.05 mIU/mL (luteinizing hormone), and 0.012 ng/mL (testosterone).

Moreover, the samples of the subjects with sex hormone concentrations less than the lower limit of detection were examined again using liquid chromatography–tandem mass spectrometry (Agilent 1200, Agilent, USA; API 4000, Applied Biosystems, Foster City, CA, USA) to obtain reliable results. The coefficients of variation were as follows: 3.8% for progesterone, 5.7% for E2, 4.4% for prolactin, 5.4% for testosterone, 5.2% for luteinizing hormone, and 6.2% for follicle-stimulating hormone.

### Statistical Analysis

The data were analyzed using SPSS13.0 (SPSS, Chicago, IL, USA) and GraphPad Prism 6 software (GraphPad, La Jolla, CA, USA). The results are presented as the mean ± standard deviation (SD). Normality was assessed using the Kolmogorov–Smirnov test. Chi-square tests were performed to compare the categorical variables between the groups. Independent-samples *t*-test and Mann–Whitney test were used to compare the continuous variables between groups. Cox proportional hazard models were used to obtain hazard ratios (HR) and to identify the baseline factors that predicted which subjects would be classified into the surviving group during the follow-up period. Patient clinical end points were calculated using the Kaplan–Meier method and compared using the log-rank test. A two-sided *P* < 0.05 was considered statistically significant.

## Results

### Characteristics of the Study Subjects

A total of 157 female subjects with hip fractures were eligible for the study. Among these, 13 (8.28%) deceased within 30 days; the following were the causes of death: lung injury and lung infection (five subjects), congestive heart failure (three subjects), postoperative infection (two subjects), renal failure (two subjects), and cerebrovascular diseases (one subject). The deceased had lower progesterone (*P* = 0.021) and E2 (*P* < 0.001) levels than the surviving group. The general and clinical characteristics of the subjects are presented in Table 1.

**Table 1:** Baseline demographic and disease characteristics of subjects.

	**Survived at 30 days (*n* = 144)**	**Deceased at 30 days (*n* = 13)**	**t/z value**	***P*-value**
Age (year)	74.06 ± 7.56	75.54 ± 6.15	0.686	0.494^&^
BMI (kg/m^2^)	22.50 ± 3.60	21.62 ± 4.06	0.837	0.404^&^
SBP (mm Hg)	122.70 ± 19.17	129.69 ± 18.77	0.643	0.521^&^
DBP (mm Hg)	70.60 ± 10.70	71.62 ± 5.25	0.337	0.737^&^
Diabetes (yes/no)	8/136	1/12	0.101	0.550^*^
Hypertension (yes/no)	16/128	2/11	0.215	0.647^*^
Follow up period (days)	30.00 ± 0.00	10.85 ± 8.03	17.455	<0.001^&^
Prolactin (ng/ml)	15.61 ± 9.70	20.37 ± 4.67	0.688	0.493#
Luteinizing hormone (mIU/ml)	19.94 ± 10.61	16.93 ± 7.07	0.399	0.691#
Testosterone (ng/ml)	0.54 ± 0.24	0.59 ± 0.09	0.345	0.731#
Follicle-stimulating hormone (mIU/ml)	38.72 ± 12.53	44.49 ± 7.90	0.646	0.520#
Progesterone (ng/ml)	0.90 ± 0.61	0.50 ± 0.30	2.323	0.021#
E2 (pg/ml)	27.24 ± 12.12	10.27 ± 9.49	4.851	<0.001#

&., Independent-samples t-test; #, Mann-Whitney Test;

*, *Fisher exact test; BMI, body mass index; SBP, systolic blood pressure; DBP, diastolic blood pressure; E2, 17-β-estradiol*.

### Univariate Cox Regression Analysis for 30-Day Mortality in Older Women Undergoing Hip Fracture Surgery

Univariate analysis identified older age (HR = 1.038, 95% CI = 1.09–1.101) as a risk factor and higher progesterone (HR = 0.162, 95% CI = 0.041–0.643) and E2 (HR = 0.939, 95% CI = 0.904–0.976) levels as protective factors for 30-day mortality in this study cohort (see Table 2).

**Table 2:** Factors associated with 30-day mortality using univariate Cox proportional Hazard analysis.

	**HR (95% CI)**	***p*-value**
Age (year)	1.038 (1.090–1.101)	0.021
BMI (kg/m^2^)	0.905 (0.785–1.044)	0.169
SBP (mm Hg)	1.022 (0.997–1.048)	0.087
DBP (mm Hg)	0.991 (0.949–1.035)	0.678
Diabetes	0.509 (0.068–3.825)	0.512
Hypertension	0.038 (0.000–9.808)	0.249
Prolactin (ng/ml)	1.029 (0.954–1.109)	0.461
Luteinizing hormone (mIU/ml)	0.991 (0.917–1.071)	0.825
Testosterone (ng/ml)	0.818 (0.107–3.842)	0.181
Follicle-stimulating hormone (mIU/ml)	0.966 (0.895–1.043)	0.376
Progesterone (ng/ml)	0.162 (0.041–0.643)	0.010
E2 (pg/ml)	0.939 (0.904–0.976)	0.001

*BMI, body mass index; SBP, systolic blood pressure; DBP, diastolic blood pressure; E2, 17-β-estradiol*.

### Multivariate Cox Regression Analysis for 30-Day Mortality in Older Women Undergoing Hip Fracture Surgery

After univariate analysis, multivariate Cox regression analysis was performed to analyze the association between the baseline factors and 30-day mortality in older women undergoing hip fracture surgery using two models (A and B). In model A (adjusted for age), higher progesterone (HR = 0.146, 95% CI = 0.036–0.598) and E2 (HR = 0.816, 95% CI = 0.707–0.941) levels were the key predictive and protective factors for 30-day mortality in older women undergoing hip fracture surgery (see Table 3).

**Table 3:** Factors associated with 30-day mortality using multivariate Cox proportional Hazard analysis.

**Variable**	**Model A**		**Model B**	
	**HR (95% CI)**	***P*-value**	**HR (95% CI)**	***P*-value**
Age (year)	NA	NA	1.397 (1.002–1.948)	0.049
Progesterone (ng/ml)	0.146 (0.036–0.598)	0.007	0.168 (0.037–0.673)	0.013
E2 (pg/ml)	0.816 (0.707–0.941)	0.005	0.857 (0.690–0.968)	0.019

*BMI, body mass index; SBP, systolic blood pressure; DBP, diastolic blood pressure; E2, 17-β-estradiol. Model A adjusted for age. Model B adjusted for BMI, SBP, DBP, diabetes, hypertension, prolactin, luteinizing hormone, testosterone, follicle-stimulating hormone*.

These results did not change after adjusting the data for systolic blood pressure (SBP), diastolic blood pressure (DBP), diabetes, hypertension, prolactin, luteinizing hormone, testosterone, and follicle-stimulating hormone in model B; higher progesterone (HR = 0.168, 95% CI = 0.037–0.673) and E2 (HR = 0.857, 95% CI = 0.690–0.968) levels were the key predictive and protective factors for 30-day mortality in older women undergoing hip fracture surgery (see Table 3).

### Kaplan–Meier Survival Analysis

**Figure 2:**
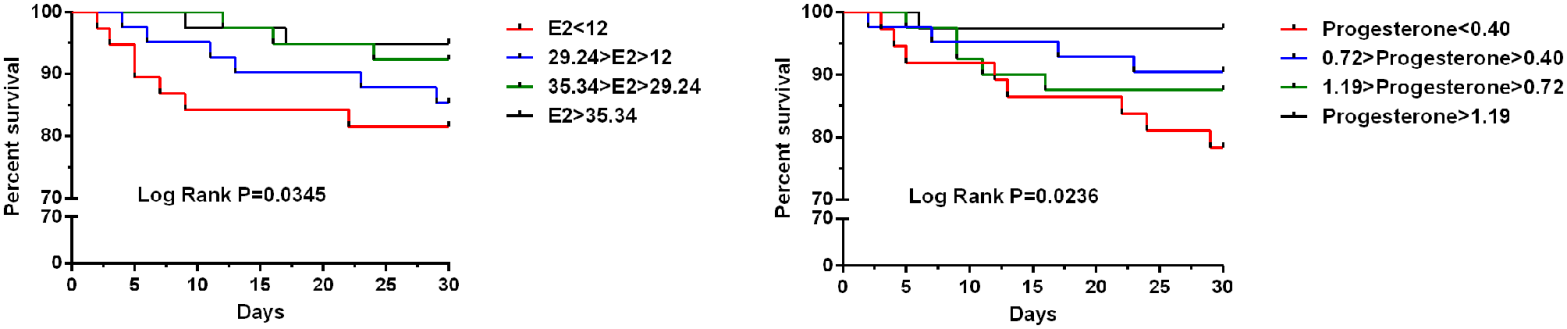
Kaplan–Meier curve stratified by E2 and progesterone according to quartiles regarding 30-day mortality for older women undergoing hip fracture surgery.

To test whether progesterone and E2 levels influence the 30-day mortality of hip fracture patients, we divided the subjects according to their quartiles of progesterone and E2 levels. The progesterone quartiles were as follows: lowest quartile (<0.40), lower quartile (0.40–0.72), higher quartile (0.72–1.19), and highest quartile (>1.19). Pairwise log-rank tests indicated significant differences between the highest quartile and the lowest, lower, and higher quartiles (*P* = 0.0345) (Figure 2).

The E2 quartiles were as follows: lowest quartile (<12), lower quartile (12–29.24), higher quartile (29.24–35.34), and highest quartile (>35.34). Pairwise log-rank tests indicated significant differences between the highest quartile and the lowest, lower, and higher quartiles (*P* = 0.0236) (Figure 2).

## Discussion

In this cohort study, we demonstrated that lower E2 and progesterone levels at the time of preoperative assessment were associated with higher risk of 30-day mortality in older women undergoing hip fracture surgery. Our multivariate Cox regression analysis showed that decreased E2 and progesterone levels are risk factors, independent from other parameters including age, SBP, DBP, diabetes, hypertension, prolactin, luteinizing hormone, testosterone, and follicle-stimulating hormone, and that they might be predictors of 30-day mortality in older women undergoing hip fracture surgery. To our knowledge, this is the first study to examine the association between mortality and E2 and progesterone levels in older women undergoing hip fracture surgery.

Demographics, injury and comorbidities, body composition, complications, and acute care are associated with the functional outcomes of hip fracture surgery (16); for example, age, male gender, inflammation, anemia, and infectious diseases foster multiple fracture hallmark functions, thus affecting patient survival. Sex hormones have been reported to be linked with inflammation, age, gender, and infectious diseases (15, 17, 18). However, the prognostic significance of sex hormones in older women undergoing hip fracture surgery remains unknown. Nevertheless, many studies support the association between higher sex hormone levels and lower risk of hip fractures (9–12). Goderie-Plomp et al. performed an age-matched, case-control study and showed that women with low estradiol levels had a 7.8 times higher risk of incident vertebral fracture (95% CI = 2.7–22.5, *P* < 0.001), adjusted for age and weight (10). Moreover, a cohort study suggested that the estrogen effect on reducing spine fracture is at least in part attributable to its effect on bone structure (11). Thus, we hypothesized that decreased sex hormone levels may lead to poor clinical outcomes in postmenopausal women undergoing hip fracture surgery.

Many efforts have been made to investigate the relationship between sex hormones and prognosis of various types of diseases. For example, Gong et al. reported that the E2 elevation group of post–abdominal surgery patients with systemic inflammatory response syndrome had a significantly shorter ICU stay and significantly lower 28-day mortality rate (19). Yu et al. showed that sex steroids are associated with better postoperative ophthalmologic outcomes in postmenopausal women undergoing vitrectomy (20). Lopez et al. performed a 9.5-year follow-up study and showed 2.60-fold increased risk of mortality among men with testosterone deficiency compared with men without testosterone deficiency, indicating that men with testosterone deficiency have a higher risk of mortality (21). In our study, lower E2 and progesterone levels at the time of preoperative assessment were associated with higher risk of 30-day mortality in older women undergoing hip fracture surgery. In contrast, Schaffrath et al. performed a large sample, population-based cohort study and found no consistent associations between sex hormones in women and incident cardiovascular diseases or mortality risk (22). The following are the possible reasons for this inconsistent result: (1) young age of subjects compared with our study population (49 vs. 70 years), (2) differences in menopausal status (only postmenopausal vs. mainly premenopausal), and (3) different study populations (general population vs. patients with hip fracture).

Research on the association of serum E2 and progesterone levels with mortality in older women undergoing hip fracture surgery is limited. In this study, survival analysis showed that the subjects with lower E2 and progesterone levels had a significantly higher percentage of 30-day mortality. This was consistent with previous results, which showed that lower E2 levels are associated with poor clinical outcomes in various types of diseases. Our results raise a critical question: What is the role of E2 and progesterone in the mortality of older women undergoing hip fracture surgery? The possible mechanism behind their role is their anti-inflammatory properties. Estrogen is associated with decreased inflammatory response because it reduces cytokine levels (15, 23, 24). Several studies have shown that 30-day mortality after a hip fracture is associated with elevated inflammatory cytokines, such as C-reactive protein, interleukin (IL)-6, IL-8, tumor necrosis factor-α, and soluble urokinase plasminogen activating receptor (8, 25). Therefore, E2 and progesterone may directly reduce cytokine levels either in the hipbone alone or throughout the body to improve the prognosis of older women undergoing hip fracture surgery. Furthermore, sex steroid hormones play a critical role in normal bone development and maintenance (26). Most prospective studies reported that E2 is the best independent predictor of bone loss (27, 28). Therefore, sex steroid hormones may likely contribute to the prognosis of older women undergoing hip fracture surgery by regulating bone growth and fracture union.

Our study has several limitations. First, single-center cohort studies, in general, have an inherent bias. Therefore, additional prospective, multicenter cohort studies are necessary to validate our findings. Second, the treatments received by the subjects may have possibly influenced the outcomes. Thus, the results may have been affected either by behavioral changes in subjects consequent to the knowledge of their disease status or by any kind of treatment. Finally, nutritional issue may be a confounding factor; however, we were unable to evaluate this factor in our study.

In conclusion, this study shows that lower E2 and progesterone levels are associated with higher risk of 30-day mortality after hip fracture surgery in older women and might be novel markers for the prognosis of 30-day mortality. Further studies should attempt to illuminate the mechanism of these hormones and confirm their role in different population groups.

## Data Availability Statement

The raw data supporting the conclusions of this article will be made available by the authors, without undue reservation.

## Ethics Statement

The studies involving human participants were reviewed and approved by the Ethics Committee of the Shanghai Xuhui Central Hospital. The patients/participants provided their written informed consent to participate in this study. Written informed consent was obtained from the individual(s) for the publication of any potentially identifiable images or data included in this article.

## Author Contributions

YZ, ZX, and JC conceived the study and participated in drafting the final manuscript. YZ, ZX, JZ, JT, FL, YS, and JC analyzed the data and completed the final draft of the manuscript. YZ and ZX prepared all the figures. All authors contributed to the article and approved the submitted version.

## Conflict of Interest

The authors declare that the research was conducted in the absence of any commercial or financial relationships that could be construed as a potential conflict of interest.
